# The relationship of body image with sexual dysfunction and dyadic adjustment in individuals diagnosed with bipolar disorder in Türkiye

**DOI:** 10.3389/fpsyt.2025.1515677

**Published:** 2025-02-26

**Authors:** Emine Reyhan Yazar, Filiz İzci

**Affiliations:** ^1^ Department of Mental Health and Diseases, Adana City Training and Research Hospital, Adana, Türkiye; ^2^ Department of Mental Health and Diseases, Istanbul Erenköy Mental and Neurological Diseases Hospital, İstanbul, Türkiye

**Keywords:** bipolar disorder, sexual dysfunction, dyadic adjustment, body image, quality of life

## Abstract

**Introduction:**

The objective of this study is to evaluate the relationship of body image (BI) with sexual dysfunction (SD) and dyadic adjustment (DA) in individuals diagnosed with Bipolar Disorder (BD). Dyadic adjustment has been defined as a dynamic process determined by variables such as relationship differences, interpersonal tensions and personal concerns, relationship satisfaction, and couple consensus. In this context, it was specifically aimed to determine the link between these factors and quality of life (QoL) in individuals with BD based on the effects of BI on sexual function and marital adjustment.

**Methods:**

This study was conducted in a psychiatric hospital in Turkiye. The population of this cross-sectional, single-center study consisted of 110 individuals who applied to the outpatient clinics of Istanbul Erenköy Mental Health and Neurological Diseases Training and Research Hospital between March 2020 and August 2020. The Structured Clinical Interview for the Diagnostic and Statistical Manual of Mental Disorders, Fifth Edition (DSM-5)Disorders-Clinician Version (SCID-5-CV), sociodemographic and clinical data form, Young Mania Rating Scale (YMRS), Hamilton Depression Rating Scale (HAM-D), Golombok-Rust Inventory of Sexual Satisfaction (GRISS), Dyadic Adjustment Scale (DAS), Body Image Scale (BIS) and the Brief Quality of Life in Bipolar Disorder (Brief QoL.BD) Questionnaire were used to collect the research data.

**Results:**

The study sample consisted of 80 individuals, 50 females and 30 males, who were diagnosed with BD and were in remission. The rate of SD in individuals with BD was found to be 55%. The most common SD was vaginismus in female individuals with BD and premature ejaculation in male individuals with BD. It has been observed that SD negatively affected DA in individuals with BD. On the other hand, no significant relationship was found between body mass index (BMI) and BI or between BI and SD in individuals with BD. However, increased satisfaction with BI positively affected DA and QoL.

**Discussion:**

The study’s findings indicated a significant relationship between sexual satisfaction, marital adjustment, BI, and QoL in individuals with BD. While no significant change was observed in DA in male individuals with BD, it was found that sexual satisfaction decreased as DA deteriorated in female individuals with BD. Additionally, it was found that BI affected DA in individuals with BD but not sexual satisfaction. Lastly, no significant relationship was observed between BMI and BI, DA, or QoL.

## 1 Introduction

Most mental disorders begin in individuals’ late adolescence and early adulthood and coincide with their sexual developmental stages. Sexual dysfunction (SD) is a broad term that includes disorders of sexual desire and arousal, erectile dysfunction, genital or pelvic pain, penetration problems, orgasm, and ejaculation disorders. Although SD is common in individuals with mental disorders, it is often overlooked (1, 2). Studies conducted with individuals diagnosed with bipolar disorder (BD) have shown that the rate of SD is higher in individuals with BD compared to healthy individuals (3). It has been reported that among individuals with psychiatric disorders, SD is most commonly seen among individuals with BD (4). While increased libido, inappropriate sexual relations, and hypersexuality are frequently observed in manic and hypomanic episodes of BD (5), loss of sexual desire and anhedonia come to the fore in depressive episodes. Therefore, the effects of manic and depressive episodes on sexual functions in BD are opposite (6).

As mentioned in a study conducted in Türkiye, one of the most important factors affecting sexual functions is dyadic adjustment (DA). It has been suggested that there is a reciprocal interaction between DA and sexuality (7). Individuals with BD often experience deterioration in their relationships as couples due to SD and mood swings. Hypersexuality, especially during manic and hypomanic episodes, is one of the most well-known symptoms of BD. Similarly, the cycle of manic and depressive episodes can create a stressful and difficult process for both the individual with BD and his/her partner, leading to imbalances in sexual functions (6). Body weight and body image (BI) are also important factors affecting sexual life. Sexual life is considered one of the determinants of quality of life (QoL). In this regard, sexual functions are also an important indicator of general functionality (8). SD is a common persistent symptom in individuals with BD in remission and has more negative effects on their QoL than depressive symptoms (9).

In view of the foregoing, this study was carried out to evaluate the relationship of BI with SD and DA in individuals with BD. In this context, it was specifically aimed to determine the link between the emotional and psychosocial effects of BD and the sexual functions of individuals with BD and their relationships with their partners. Our study aims to provide a deeper understanding of how sexual dysfunctions and DA are affected in individuals diagnosed with BD by revealing the role of BI on these two basic psychosocial parameters.

### 1.1 Hypothesis of the study

Hypothesis 1: Negative body image is associated with sexual dysfunction in patients with Bipolar Disorder.
Hypothesis 2: Negative body image is associated with couple adjustment in patients with Bipolar Disorder.
Hypothesis 3: Sexual dysfunction is associated with couple adjustment in patients with Bipolar Disorder.

## 2 Materials and methods

### 2.1 Population and sample

This study was designed as a cross-sectional, single-center study. The study population consisted of 110 individuals with BD who applied to the outpatient clinics of Istanbul Erenköy Mental Health and Neurological Diseases Training and Research Hospital in Türkiye between March 2020 and August 2020. The study was approved by Erenköy Mental and Neurological Diseases Education and Research Hospital Clinical Research Ethics Committee, with a protocol number of 4 and date as 03.02.2020. Sixteen individuals with BD were excluded from the study because they did not continue to attend the interviews, six were not in remission, and eight did not fill out all the data collection instruments administered. In the end, 80 individuals with BD who agreed to participate in the study and gave their consent were included in the study.

BD diagnoses were confirmed using the Structured Clinical Interview for the Diagnostic and Statistical Manual of Mental Disorders, Fifth Edition (DSM-5) Disorders-Clinician Version (SCID-5-CV). A sociodemographic and clinical data form was also filled out for each individual with BD, and whether individuals with BD were in remission was determined by their Hamilton Depression Rating Scale (HAM-D) scores being 8 or less and their Young Mania Rating Scale (YMRS) scores being 6 or less. The last episode occurring at least three months ago was also accepted as one of the remission criteria. Additionally, individuals with mental developmental delay, neurocognitive disorder, brain injury, alcohol and/or substance use disorder history, and those who had received electroconvulsive therapy (ECT) within the last six months were also excluded from the study.

All participants were administered the Golombok-Rust Inventory of Sexual Satisfaction (GRISS), Dyadic Adjustment Scale (DAS), Body Image Scale (BIS), and the Brief Quality of Life in Bipolar Disorder (Brief QoL.BD) Questionnaire self-report scales.

### 2.2 Data collection tools

#### 2.2.1 Sociodemographic and clinical data form

A sociodemographic and clinical data form was developed by the researchers in accordance with the objectives of the study and was filled out during the first interview with the participants to obtain information about their health status and medical history. The form included questions regarding age, gender, marital status, employment status, education level, place of residence, socioeconomic status, and sexual functions.

#### 2.2.2 The structured clinical interview for DSM-5 disorders-clinician version

SCID-5-CV, developed by First et al. (10), is a structured clinical tool administered by the interviewer to make a clinical diagnosis based on DSM-V diagnostic criteria. The adaptation of SCID-5-CV to Turkish and the validity and reliability studies of the Turkish version of SCID-5-CV were conducted by Aydemir et al. (11).

#### 2.2.3 Hamilton depression rating scale

The 17-item HAM-D, developed by Hamilton, is a structured tool administered by the interviewer to assess the degree and severity of depression in the individual and is administered by the interviewer (12). Each item is assigned a score between 0 and 4. Accordingly, the highest score that can be obtained from HAM-D is 51. The validity and reliability studies of the Turkish version of HAM-D were conducted by Akdemir et al. (13).

#### 2.2.4 Young mania rating scale

The 11-item YMRS, developed by Young et al., is a structured tool administered by the interviewer to assess the severity of the manic state (14). The highest score that can be obtained from the YMRS is 60. Validity and reliability studies of the Turkish version of YMRS were conducted by Karadağ et al. (15).

#### 2.2.5 Golombok-rust inventory of sexual satisfaction

The 28-item GRISS, developed as a Likert-type tool by Rust and Golombok in 1985, is used to evaluate the quality of sexual intercourse in heterosexual men and women and sexual functions and SD in individuals with heterosexual sexual life (16). The adaptation and standardization of GRISS to Turkish was carried out by Tuğrul et al. in 1995 (17).

#### 2.2.6 Dyadic adjustment scale

The 32-item DAS, developed by Spanier in 1976 as a Likert-type tool, is used to assess the marital adjustment of married or cohabiting couples (18). DAS includes four sub-dimensions: dyadic satisfaction, dyadic cohesion, dyadic consensus, and affective expression. The higher the DAS total score, the greater the DA. The validity and reliability studies of the Turkish version of DAS were conducted by Fışılıoğlu and Demir in 2000 (19).

#### 2.2.7 Body image scale

The 40-item BIS, developed by Secord and Jourard in 1953, is used to assess individuals’ BI (20). The higher the total BIS score, the higher the satisfaction with BI. Individuals with a BIS total score below the cut-off value of 135 are defined as having low BI. The adaptation of BIS to Turkish and the validity and reliability studies of the Turkish version of BIS were conducted by Hovardaoğlu in 1989 (21).

#### 2.2.8 Brief quality of life in bipolar disorder questionnaire

The 12-item Brief QoL. BD was developed by Michalak and Murray in Canada in 2010 (22). Each item assesses satisfaction with QoL over the last seven days. The total score that can be obtained from the Brief QoL. BD varies between 12 and 60. The higher the total Brief QoL. BD score, the higher the QoL (22). Validity and reliability studies of the Turkish version of Brief QoL. BD were conducted by Gümüş et al. (23).

### 2.3 Statistical analysis

Statistical analyses of the collected data were performed using SPSS Statistics 17.0 (Statistical Package for the Social Sciences for Windows, Version 17.0, SPSS Inc., Chicago, IL, US, 2008) software package. The normal distribution characteristics of variables were analyzed using histogram graphs and the Kolmogorov-Smirnov test. The results of the statistical analyses of the research data were expressed using descriptive statistics, including mean ± standard deviation and median values. Mann-Whitney U test was used to compare the differences in variables determined not to conform to the normal distribution between the two groups. Spearman’s correlation test was used to evaluate the relationships between the variables. Probability (*P*) statistics of < 0.05 were deemed to indicate statistical significance.

## 3 Results

### 3.1 Sociodemographic characteristics of the sample

The mean age of the sample, 50 females and 30 males, was 42.93 ± 9.70 years. The mean age of the female participants was 41.10 ± 8.36 years, and that of male participants was 45.97 ± 11.09 years.

In terms of education level, the number of participants who were literate or elementary school graduates was 32 (49%), the number of those who were middle school graduates was 14 (17.5%), the number of those who were high school graduates was 22 (27.50%) and the number of those who graduated from a vocational school or a university was 12 (15%). In terms of their employment status, the number of participants who were actively employed was 26 (32.5%). In terms of the method of meeting the spouse, the number of participants who got married by dating was 33 (41.25%), and the number of those who got married by arranged marriage was 47 (58.75%). In terms of average monthly income, the number of participants with an income of 1500 Turkish Liras (TL) or less was 5 (6.25%), the number of those with an income of 3000 TL or more was 37 (46.25%), and the number of those with an income between 1500 TL and 3000 TL was 38 (47.50%). The majority of participants (95%) were married. Of these, 72 (90%) were in their first marriage, 7 (8.75%) were in their second marriage, and 1 (1.25%) was in his/her third marriage. The number of participants who had a child or children was 68 (85%), and the number of those who have been smoking was 33 (41.25%). There was no one in the sample with alcohol or substance use (**Table 1**).

**Table 1:** Sociodemographic characteristics of individuals with bipolar disorder.

	Mean ± SD/n	Median/%
Age*	42.93 ± 9.70	42
BMI*	30.11 ± 5.13	29.9
Gender		
Male	30	37.5
Female	50	62.5
Education level		
Literature/Elementary school graduate	32	40
Middle school graduate	14	17.5
High school graduate	22	27.5
Vocational school or university graduate	12	15
Employment status		
Employed	26	32.5
Unemployed	54	67.5
Method of meeting the spouse		
Dating	33	41.25
Arranged marriage	47	58.75
Average monthly income		
1500 TL or less	5	6.25
between 1500TL and 3000 TL	38	47.5
3000 TL or more	37	46.25
Marital status		
Married	76	95
Single	4	5
Number of marriages		
1	72	90
2	7	8.75
3	1	1.25
Participants with a child/child	68	85
Participants who have been smoking	33	41.25

*Mean ± Standard Deviation (SD) and Median.

The participants’ mean body mass index (BMI) was calculated as 30.11 ± 5.13.

### 3.2 Assessment of the correlations between GRISS and DAS scores

The correlations between DAS and GRISS total and subscale scores were assessed separately for female and male participants. Accordingly, the DAS total score was negatively correlated with GRISS communication, satisfaction, and touch subscale scores in male participants (r = -0.390, *P* < 0.05; r = -0.363, *P* < 0.05; and r = -0.433, *P* < 0.05, respectively), and with GRISS total score and GRISS frequency, satisfaction, avoidance and touch subscale scores in female participants (r = -0.536, *P* < 0.01; r = -0.381, *P* < 0.01; r = -0.550, *P* < 0.01; r = -0.435, *P* < 0.01; and r = -0.449, *P* < 0.01, respectively). DAS dyadic satisfaction subscale score was negatively correlated with GRISS satisfaction subscale score in male participants (r = -0.422, *P* < 0.05) and with GRISS total score and GRISS satisfaction, avoidance, and touch subscale scores (r = -0.557, *P* < 0.01; r = -0.449, *P* < 0.01; r = -0.508, *P* < 0.01; and r = -0.460, *P* < 0.01, respectively). DAS dyadic consensus subscale score was negatively correlated with GRISS communication and touch subscale scores in male participants (r = -0.381, *P* < 0.05and r = -0.399, *P* < 0.05, respectively), and with GRISS total score and GRISS frequency, satisfaction, avoidance and touch subscale scores in female participants (r = -0.427, *P* < 0.01; r = -0.346, *P* < 0.05; r = 0.467, *P* < 0.01; r = -0.353, *P* < 0.05; and r = -0.333, *P* < 0.05, respectively). DAS dyadic cohesion subscale score was negatively correlated with the GRISS communication subscale score in male participants (r = -0.458, *P* < 0.05) and with the GRISS total score and GRISS satisfaction and touch subscale scores in female participants (r = -0.424, *P* < 0.01; r = -0.362, *P* < 0.05; and r = -0.318, *P* < 0.05, respectively). DAS affective expression subscale score was negatively correlated with GRISS total score and GRISS communication and touch subscale scores in male participants(r = -0.496, *P* < 0.01; r = -0.386, *P* < 0.05; and r = -0.667, *P* < 0.01, respectively), and with GRISS total score and GRISS frequency, satisfaction and touch subscale scores in female participants (r = -0.495, *P* < 0.01; r = -0.370, *P* < 0.01; r = -0.429, *P* < 0.01; and r = -0.381, *P* < 0.01, respectively) (**Table 2**).

**Table 2:** Correlations between GRISS and DAS Total and Subscale Scores in individuals with bipolar disorder.

			DAS															
			Total			Dyadic Satisfaction			Dyadic Consensus			Dyadic Cohesion			Affective Expression		
			Overall	Males	Females	Overall	Males	Females	Overall	Males	Females	Overall	Males	Females	Overall	Males	Females
GRISS	Total	r	-0.467	-0.358	-0.536	-0.441	-0.351	-0.557	-0.389	-0.238	-0.427	-0.332	-0.184	-0.424	-0.485	-0.496	-0.495
		p	0.000*	0.052	0.000*	0.000*	0.057	0.000*	0.000*	0.206	0.002*	0.003*	0.331	0.002*	0.000*	0.005*	0.000*
	Frequency	r	-0.264	-0.07	-0.381	-0.179	-0.068	-0.221	-0.229	0.025	-0.346	-0.116	0.06	-0.194	-0.313	-0.189	-0.37
		p	0.018**	0.714	0.006*	0.114	0.721	0.127	0.042**	0.894	0.015**	0.31	0.754	0.182	0.005*	0.318	0.009*
	Communication	r	-0.164	-0.39	0.014	-0.158	-0.203	-0.063	-0.063	-0.381	0.086	-0.23	-0.458	-0.026	-0.245	-0.386	-0.137
		p	0.145	0.033**	0.921	0.165	0.283	0.668	0.583	0.038	0.559	0.041**	0.011**	0.86	0.030**	0.035**	0.348
	Satisfaction	r	-0.483	-0.363	-0.55	-0.457	-0.422	-0.449	-0.385	-0.26	-0.467	-0.267	-0.113	-0.362	-0.404	-0.338	-0.429
		p	0.000*	0.049**	0.000*	0.000*	0.020**	0.001*	0.000*	0.166	0.001*	0.017**	0.553	0.011**	0.000*	0.068	0.002*
	Avoidance	r	-0.303	-0.042	-0.435	-0.421	-0.179	-0.508	-0.208	0.046	-0.353	-0.171	0.072	-0.262	-0.258	-0.069	-0.28
		p	0.006*	0.825	0.002*	0.000*	0.345	0.000*	0.065	0.811	0.013**	0.133	0.706	0.068	0.022**	0.717	0.052
	Touch	r	-0.442	-0.433	-0.449	-0.441	-0.313	-0.46	-0.321	-0.399	-0.333	-0.334	-0.251	-0.318	-0.465	-0.667	-0.381
		p	0.000*	0.017**	0.001*	0.000*	0.092	0.001*	0.004*	0.029**	0.019**	0.003*	0.181	0.026**	0.000*	0.000*	0.007*
	Impotence	r		-0.167			-0.201			-0.057			-0.052			-0.179	
		p		0.377			0.287			0.764			0.784			0.344	
	Premature ejaculation	r		-0.237			-0.205			-0.248			-0.145			-0.288	
		p		0.208			0.276			0.186			0.443			0.123	
	Vaginismus	r			-0.281			-0.293			-0.277			-0.198			-0.36
		p			0.048**			0.041**			0.054			0.173			0.011**
	Anorgasmia	r			-0.445			-0.412			-0.358			-0.453			-0.348
		p			0.001*			0.003*			0.012**			0.001*			0.014**

Spearman’s correlation test, *P* < 0.01 *, *P* < 0.05**.

### 3.3 Assessment of the correlations between BIS and Brief QoL.BD Scores and DAS and GRISS scores

The correlations between BIS and Brief QoL. BD scores and DAS and GRISS scores were assessed separately for female and male participants. Accordingly, there was a positive correlation between the BIS score and the DAS dyadic consensus subscale score (r = 0.434, *P* < 0.05) and positive correlations between Brief QoL. BD score and DAS total score and DAS dyadic consensus, dyadic cohesion, and affective expression subscale scores (r = 0.381, *P* < 0.05; r = 0.411, *P* < 0.05; r = 0.376, *P* < 0.05; and r = 0.528, *P* < 0.01, respectively) in male participants. Similarly, there were positive correlations between BIS score and DAS total score and DAS dyadic satisfaction and dyadic consensus subscale scores (r = 0.375, *P* < 0.01; r = 0.320, *P* < 0.05; and r = 0.311, *P* < 0.05, respectively), and between BIS score and GRISS avoidance and touch subscale scores (r = -0.376, *P* < 0.01and r = -0.330, *P* < 0.05, respectively) in female participants (**Table 3**).

**Table 3:** Correlations between BIS, Brief QoL.BD, DAS, and GRISS Scores in male and female participants.

	Males				Females			
	BIS		Brief QoL.BD		BIS		Brief QoL.BD	
	r	p	r	p	r	p	r	p
DAS-Total	0.195	0.301	0.381	0.038**	0.375	0.008*	0.209	0.150
DAS -Dyadic satisfaction	-0.069	0.719	0.128	0.500	0.320	0.025**	0.123	0.399
DAS- Dyadic consensus	0.434	0.016**	0.411	0.024**	0.311	0.029**	0.125	0.391
DAS- Dyadic cohesion	0.219	0.244	0.376	0.040**	0.182	0.210	0.082	0.575
DAS–Affective expression	0.222	0.238	0.528	0.003*	0.175	0.230	0.244	0.091
GRISS- Total	0.046	0.808	-0.293	0.116	-0.206	0.155	-0.263	0.068
GRISS-Frequency	-0.143	0.452	-0.028	0.884	-0.183	0.209	-0.273	0.058
GRISS-Communication	0.046	0.810	-0.153	0.419	0.030	0.839	-0.242	0.094
GRISS-Satisfaction	0.007	0.970	-0.027	0.886	-0.204	0.160	-0.227	0.117
GRISS-Avoidance	-0.040	0.836	-0.066	0.728	-0.376	0.008*	-0.045	0.757
GRISS-Touch	-0.037	0.847	-0.306	0.100	-0.330	0.021**	-0.260	0.071

Spearman’s correlation test, *P* < 0.01 *P < 0.05**.

### 3.4 Assessment of the correlations between GRISS total, DAS total, BIS, and Brief QoL. BD scores

The correlations between GRISS total, DAS total, BIS, and Brief QoL. BD scores were assessed for the overall sample. Accordingly, the GRISS total score was negatively correlated with the DAS total and Brief QoL.BD scores (r = -0.467, *P* < 0.01 and r = -0.302, *P* < 0.01, respectively). On the other hand, there was no significant correlation between the GRISS total score and the BIS score. DAS total score was positively correlated with BIS and Brief QoL.BD scores (r = 0.334, *P* < 0.01 and r = 0.344, *P* < 0.01, respectively). There was also a positive correlation between BIS and Brief QoL. BD scores (r = 0.344, *P* < 0.01) (**Table 4**).

**Table 4:** Correlations between GRISS Total, DAS Total, BIS, and Brief QoL.BD Scores in individuals with bipolar disorder.

	GRISS-Total		DAS-Total		BIS		Brief QoL.BD	
	r	p	r	p	r	p	r	P
GRISS-Total			-0.467	0.000*	-0.097	0.394	-0.302	0.007*
DAS- Total	-0.467	0.000*			0.334	0.003*	0.278	0.013**
BIS	-0.097	0.394	0.334	0.003*			0.344	0.002*
Brief QoL.BD	-0.302	0.007*	0.278	0.013**	0.344	0.002*		

Spearman’s correlation test, *P* < 0.01 *P < 0.05**.

### 3.5 Assessment of the correlations between BMI and DAS, BIS, and Brief QoL. BD scores

No significant correlation was found between BMI and DAS, BIS, and Brief QoL. BD scores (r = -0.067, *P* = 0.553; r = -0.189, *P* = 0.096; and r = 0.018, *P* = 0.875, respectively) (**Table 5**).

**Table 5:** Correlations between BMI, DAS, BIS, and Brief QoL.BD Scores in individuals with bipolar disorder.

		DAS-Total	DAS Dyadic satisfaction	DAS Dyadic consensus	DAS Dyadic cohesion	DAS Affective expression	BIS	Brief QoL.BD
BMI	R	-0.067	-0.07	-0.053	-0.019	0.017	-0.189	0.018
	P	0.553	0.541	0.645	0.867	0.88	0.096	0.875

Spearman’s correlation test.

### 3.6 Drug use characteristics of the sample

There were 73 (91.25%) patients using mood stabilizers. 28 (35%) patients using lithium, 45 (56.25%) patients using valproic acid, 3 (3.25%) patients using carbamazepine, 6 (7.50%) patients using lamotrigine. 58 (72.50%) patients using atypical antipsychotics and 7 (8.75%) patients using antidepressants (**Table 6**).

**Table 6:** Drug use characteristics of the sample.

	n	%
Mood stabilizer	73	91,25
Lithium	28	35,00
Valproic acid	45	56,25
Carbamazepine	3	3,75
Lamotrigine	6	7,50
Atypical antipsychotic	58	72,50
Antidepressant	7	8,75

### 3.7 Assessments of the relationship between antidepressant use and GRISS, DAS, BAS and Brief QoL.BD scores

GRISS Total, Frequency, Avoidance and Touch values ​​of those using antidepressants were higher than those not using antidepressants (p=0.022; p=0.01; p=0.033; p=0.033, respectively) (**Table 7**).

**Table 7:** Relationship Between Antidepressant Use and GRISS, DAS, BIS and Brief QoL.BD Values.

	Antidepressant						P
	Not using antidepressants			Using antidepressants			
	Mean	s.d.	Median	Mean	s.d.	Median	
GRISS-Total	4,66	± 2,11	5,00	6,57	± 1,90	7,00	0,022**
GRISS-Frequency	4,73	± 1,92	5,00	6,57	±,98	7,00	0,010**
GRISS-Communication	4,34	± 2,36	4,00	5,00	± 3,21	5,00	0,612
GRISS-Satisfaction	3,27	± 1,54	3,00	4,86	± 2,34	4,00	0,082
GRISS-Avoidance	3,89	± 2,22	4,00	5,86	± 1,57	6,00	0,032**
GRISS-Touch	3,89	± 2,32	4,00	5,86	± 2,04	6,00	0,033**
GRISS-Impotence	4,41	± 1,72	5,00	6,00	±.	6,00	0,259
GRISS-Premature ejaculation	5,55	± 1,84	6,00	3,00	±.	3,00	0,141
GRISS-Vaginismus	5,68	± 1,75	6,00	7,00	± 1,55	7,00	0,098
GRISS-Anorgasmia	3,61	± 1,60	4,00	3,83	±,98	3,50	0,626
DAS-Total	110,10	± 20,90	115,00	93,43	± 35,04	98,00	0,261
DAS-Dyadic satisfaction	35,43	± 7,93	36,00	32,86	± 12,24	33,00	0,704
DAS-Dyadic consensus	52,01	± 10,80	53,00	43,00	± 12,99	43,00	0,065
DAS-Cohesion	13,87	± 4,20	13,50	10,86	± 7,29	12,00	0,401
DAS-Affective expression	8,94	± 2,49	9,50	6,57	± 4,31	8,00	0,114
BIS	147,40	± 27,38	147,00	130,86	± 34,14	122,00	0,140
Brief QoL.BD	43,83	± 10,70	46,00	34,14	± 14,67	29,00	0,094

Mann Whitney U Testi p<0,05**.

## 4 Discussion

Considering that scores of 5 and above indicate deterioration of sexual relations or sexual functions, GRISS premature ejaculation subscale scores in male participants and GRISS vaginismus and frequency subscale scores in female participants indicated significant impairments in these areas. Along these lines, another study conducted in our country examining sexual functions in individuals with BD reported significant impairments in females based on GRISS touch, satisfaction, and anorgasmia subscale scores and in males based on GRISS premature ejaculation, impotence, and satisfaction subscale scores (24). As in our study, Hariri et al. found significant deteriorations in GRISS premature ejaculation subscale scores in males and in GRISS vaginismus subscale scores in females (25). In a study examining sexual dysfunction in patients with severe mental illness, a significant difference was found only in the ability to reach orgasm in those with bipolar disorder (26). Additionally, a study comparing SD and DA of individuals with BD with healthy control subjects in 2015 found significant deteriorations in GRISS frequency and premature ejaculation subscale scores in males and in GRISS communication subscale scores in females (7).

In our study, the erectile dysfunction rate was found to be 63.3%. In a study conducted on sexual functions in patients diagnosed with BD with a large sample size in our country, erectile dysfunction was found to be 52% in male patients (27).In another study, erectile dysfunction was found in a small group of bipolar male patients (n = 29) at a rate similar to healthy controls (25). In a study investigating the effects of typical and atypical antipsychotic drugs on SD in patients with BD in remission, erectile dysfunction was present in 42% of the sample population and was the most common sexual dysfunction reported, and this was significantly higher in typical than atypical antipsychotics (28). The reason for the higher rate of erectile dysfunction in our study may be that a higher age group was included in this study. In addition, although the rates were similar in our study, premature ejaculation was observed more frequently than erectile dysfunction in male patients (66.67%).

In our study, the significant relationship between the use of antidepressant medication and the GRISS total score supports the relationship between sexual dysfunction and antidepressant medication. The relationship between the presence of problems in the GRISS frequency, avoidance and touch areas and antidepressant medication supports the decrease in sexual desire due to the use of antidepressant medication, in line with the literature. When we look at the literature, it has been shown that medications used in the treatment of depression can reduce sexual desire and cause deterioration in sexual functions (29).

In a study investigating sexual dysfunction due to antidepressant drugs, sexual dysfunction due to antidepressant use was found in the range of 25-80%, and the most common disorders in the sexual response cycle were observed in the stages of desire, arousal and orgasm (30).

In our study, no significant relationship was found between the use of mood stabilizers and GRISS, DAS, BIS and Brief QoL.BD values.

The varying rates of data on sexual dysfunction may be due to heterogeneous reasons such as whether the partner is questioned about the presence of sexual problems, differences between scales, duration of illness, and medical problems that accompany the person and may lead to sexual dysfunction.

We assessed the relationships between GRISS and DAS total scores and subscale scores. Consequently, we found a negative relationship between GRISS and DAS total scores in female participants, but we did not find any significant relationship between GRISS and DAS total scores in male participants. As for the relationships between GRISS and DAS subscale scores, while we found a negative relationship only between DAS satisfaction and GRISS satisfaction subscale scores in male participants, we found negative relationships between DAS satisfaction subscale score and GRISS satisfaction, avoidance, touch, anorgasmia, and vaginismus subscale scores in female participants. Notably, there was no significant relationship between GRISS frequency and communication subscale scores and DAS dyadic satisfaction subscale scores in both male and female participants. In parallel, the limited studies available in the literature on the impact of SD reported that SD accompanied dissatisfaction and negative DA in individuals with BD (7, 31). In a study investigating sexual satisfaction and marital adjustment in spouses of bipolar disorder patients, more sexual dissatisfaction and less marital adjustment were observed in spouses during manic or depressive episodes of the patients (32).

We also assessed the relationships between BI, SD, and DA in individuals with BD. Relevant literature data indicate that obesity, overweightness, and eating disorders are common among individuals with BD (33, 34). A study conducted with a cohort of 356 individuals with BD found the prevalence of eating disorders in the cohort to be 5.3%. 94.7% of individuals with BD diagnosed with eating disorders were female. Of these individuals, 57.9% had bulimia nervosa and 42.1% had anorexia nervosa (35). A systematic review found that the severity of bulimia nervosa and binge eating disorder was higher in women among patients with bipolar disorder (36).

The most common eating disorder seen in individuals with BD is binge eating disorder. Although the etiological factors underlying binge eating disorder, being the most common eating disorder in individuals with BD, are not fully known, it is thought to be related to impulse control disorder and mood disorder (37). A study investigating the relationship between bipolar disorder and binge eating disorder found a strong correlation between bipolar disorder and eating disorders. This correlation was attributed to the fact that BD is more severe in patients taking medication for BD due to the side effects of some medications, which include increased food intake, and that eating disorders are underrecognized (38).

In comparison, we did not find any significant relationship between BMI and BIS score. Similarly, another study found a very weak relationship between BMI and BI, indicating that the relationship between BI and satisfaction with body weight is more complex than thought and is also linked to psychological and cultural factors (39). As a matter of fact, a study investigating the relationship between binge eating disorder and body weight and psychosocial adjustment reported that even though the mean BMI was high (25.6) in individuals with BD, their BIS scores were high (40). Additionally, there are studies showing that the relationship between BMI and female sexual function index scores is relatively weak and that there is no direct correlation (41).

A study evaluating the relationship between SD and BI in the general population using GRISS and BIS tools reported a negative correlation between the increasing GRISS total score and the BIS score (42). In the same study, the BIS score was found to be significantly correlated with the GRISS frequency, communication, avoidance, vaginismus, and anorgasmia subscale scores in females and with the GRISS frequency, communication, avoidance, and impotence subscale scores in males. Another study examining the relationship between BI and sexual satisfaction in the general population reported that BI was not a predictor of SD and that SD was more related to self-esteem than BI (43).

In comparison, we found significant relationships between GRISS avoidance and touch subscale scores and BIS scores in females. A study evaluating BI and SD in females in the general population indicated that negative BI may cause problems in sexual desire and arousal in women (44). In parallel, a study examining the relationship between body awareness and sexual arousal in women with SD reported that mental and physical sexual arousal changed with body awareness (45).

Our assessments of the correlations between BIS and DAS subscale scores revealed that the relationship between BI and DA was stronger in females than in males. In addition, we found that the BIS scores of females were lower than those of males, although not significantly. A thorough literature review did not reveal a similar study comparing the relationship between BI and DA in individuals with mood disorders. However, studies conducted with the general population report that females are more likely to be dissatisfied and anxious about their BI. These studies have concluded that females focus more on social aspects of BI, compare their appearance with others more frequently, and report higher levels of social anxiety compared to males (43).

Our assessment of the correlations between GRISS, BIS, DAS, and Brief QoL. BD scores in individuals with BD revealed a significant relationship between BIS and Brief QoL. BD scores, indicating that the level of satisfaction with BI caused an increase in QoL. In addition, we found a negative correlation between increased GRISS scores and the Brief QoL. BD scores, suggesting that SD negatively affects QoL. Furthermore, the positive correlation we found between the DAS and Brief QoL. BD scores suggest that DA improves the QoL. There are studies in the literature that support our findings that there is a positive correlation between BI, quality of sexual life, and QoL (46). In a study conducted with a range of mental disorder diagnoses, a strong correlation was found between BI and QoL, and a moderate correlation was found between positive BI and sexual satisfaction (47). Various studies emphasize that body-related experiences have important effects on human development and QoL and that BI is a central component of an individual’s experience of the world (48).

### 4.1 Limitations of the study

The primary limitation of the study is that due to its cross-sectional design, causal relationships between variables cannot be determined, and therefore the findings of the study can only be evaluated to a limited extent in terms of temporal changes or long-term effects. The secondary limitation of the study is the limited generalizability of its findings due to its single-center design. Collecting data from different regions and a larger patient group would have been more beneficial in terms of the generalizability of the findings. In addition, evaluating patients only in remission created a deficiency in revealing how findings regarding SD and DA change during other episodes of BD. Individuals who had BD, along with other psychiatric disorders, were excluded from the study, but the effects of the medications and treatment processes on sexual functions were not examined in detail. One of the limitations of the study is that it did not investigate whether individuals were initially assessed through any psychotherapy or counselling interventions to prevent or alleviate symptoms, which could be a protective factor. Furthermore, the absence of a control group made understanding the differences between individuals with BD and healthy individuals difficult. Lastly, the use of self-report measures might have introduced bias in data in data regarding sexual function and BI, which are particularly sensitive topics. Future studies could be designed to be more comprehensive, considering these limitations.

## 5 Conclusion

This study conducted in Türkiye, the frequency of SD in individuals with BD and its relationships with DA, BI and quality of life were analyzed in depth. The findings indicated that SD was more prevalent in female individuals with BD than in male individuals with BD. Female individuals with BD experienced significant difficulties mostly in areas such as sexual satisfaction, touch, and anorgasmia, whereas male individuals with BD experienced significant difficulties mostly in areas such as premature ejaculation and impotence. DA was found to be closely related to sexual satisfaction, especially in female individuals with BD, and that as DA decreases, sexual satisfaction decreases significantly. In addition, BI was found to have a significant effect on DA but not on sexual satisfaction. It was found that BI was positively correlated with QoL and that satisfaction with BI directly affects QoL. On the other hand, no significant relationship was found between BMI and BI, DA, and QoL. In conclusion, SD in individuals with BD negatively affects not only sexual life but also general QoL. These findings indicate that individuals with BD need to be supported more in terms of SD and DA.

## Data Availability

The original contributions presented in the study are included in the article/supplementary material. Further inquiries can be directed to the corresponding author.

## References

[1] McCabe MP Sharlip ID Atalla E Balon R Fisher AD Laumann E. Definitions of sexual dysfunctions in women and men: A consensus statement from the fourth international consultation on sexual medicine 2015. J Sex Med. (2016) 13:135–43. doi: 10.1016/j.jsxm.2015.12.019 26953828

[2] Macdonald S Halliday J MacEwan T Sharkey V Farrington S Wall S. Nithsdale Schizophrenia Surveys 24: sexual dysfunction. Case-control study. Br J Psychiatry. (2003) 182:50–6. doi: 10.1192/bjp.182.1.50 12509318

[3] Arslan M Çalişkan AM Göktaş D Inanli I Çalişir S Eren I. Sexual functions in male patients with bipolar disorder and their healthy spouses. Alpha Psychiatry. (2019) . 20:245–52. doi: 10.5455/apd.5397

[4] Grover S Nehra R Thakur A. Bipolar affective disorder and its impact on various aspects of marital relationship. Ind Psychiatry J. (2017) 26:114–20. doi: 10.4103/ipj.ipj_15_16 PMC6058431 30089956

[5] Mazza M Harnic D Catalano V Di Nicola M Bruschi A Bria P. Sexual behavior in women with bipolar disorder. J Affect Disord. (2011) 131:364–7. doi: 10.1016/j.jad.2010.11.010 21130498

[6] Kopeykina I Kim HJ Khatun T Boland J Haeri S Cohen LJ. Hypersexuality and couple relationships in bipolar disorder: A review. J Affect Disord. (2016) 195:1–14. doi: 10.1016/j.jad.2016.01.035 26851616

[7] Namlı Z Karakuş G Tamam L Demirkol ME. Sexuality and sexual dysfunctions in bipolar disorder. Curr App Psychiatry. (2016) 8:309–20. doi: 10.18863/pgy.253437

[8] Poggiogalle E Di Lazzaro L Pinto A Migliaccio S Lenzi A Donini LM. Health-related quality of life and quality of sexual life in obese subjects. Int J Endocrinol. (2014) 2014:847871. doi: 10.1155/2014/847871 24707290 PMC3953417

[9] Samalin L de Chazeron I Vieta E Bellivier F Llorca PM. Residual symptoms and specific functional impairments in euthymic patients with bipolar disorder. Bipolar Disord. (2016) 18:164–73. doi: 10.1111/bdi.12376 26946486

[10] Williams MB Karg RS Spitzer RL. Structured clinical interview for DSM-5–research version (SCID-5 for DSM-5, research version; SCID-5–RV). Arlington, VA: American Psychiatric Association (2015) p. 1–94.

[11] Aydemir Ö Öztekin S Akdeniz F. Reliability and validity study of the Turkish version of Bipolar Prodrome Symptom Scale. Turkish J Psychiatry. (2018) 29:116. doi: 10.5080/u18399 30215840

[12] Hamilton M. Development of a rating scale for primary depressive illness. Br J Soc Clin Psychol. (1967) 6:278–96. doi: 10.1111/j.2044-8260.1967.tb00530.x 6080235

[13] Akdemir A Örsel DS Dağ İ Türkçapar MH İşcan N Özbay H. Validity-reliability and clinical use of the Hamilton depression rating scale (HDRS). J Psychiatry Psychol Psychopharmacol. (1996) 4:251–9.

[14] Young RC Biggs JT Ziegler VE Meyer DA. A rating scale for mania: reliability, validity and sensitivity. Br J Psychiatry. (1978) 133:429–35. doi: 10.1192/bjp.133.5.429 728692

[15] Karadağ F Oral ET Aran Yalçın F Erten E. Reliability and validity of Turkish translation of Young Mania Rating Scale. Turkish J Psychiatry. (2002) 13:107–14. 12794663

[16] Rust J Golombok S. The golombok-rust inventory of sexual satisfaction (GRISS). Br J Clin Psychol. (1985) 24:63–4. doi: 10.1111/j.2044-8260.1985.tb01314.x 3971070

[17] Tuğrul C Öztan N Kabakçı E. Standardization study of the Golombok-Rust sexual satisfaction scale. Turkish J Psychiatry. (1993) 4:83–8.

[18] Spainer GB. Dyadic adjustment scale. Toronto: Multi-Health Systems (1989).

[19] Fişiloğlu H Demir A. Applicability of the Dyadic Adjustment Scale for measurement of marital quality with Turkish couples. Eur J Psychol Assess. (2000) 16:214. doi: 10.1027/1015-5759.16.3.214

[20] Secord PF Jourard SM. The appraisal of body-cathexis: body-cathexis and the self. J Consult Psychol. (1953) 17:343–7. doi: 10.1037/h0060689 13109086

[21] Hovardaoğlu S. Body image scale. J Psychiatry Psychology Psychopharmacol (3P). (1992) 1:11–26.

[22] Michalak EE Murray G Collaborative RESearch Team to Study Psychosocial Issues in Bipolar Disorder (CREST.BD). Development of the QoL. BD: a disorder-specific scale to assess quality of life in bipolar disorder. Bipolar Disord. (2010) 12:727–40. doi: 10.1111/j.1399-5618.2010.00865.x 21040290

[23] Gümüş F Çakır S Kesebir S Michalak EE Murray G. Psychometric properties of the Turkish version of the brief quality of life in bipolar disorder (Brief QoL. BD) scale. J Psychiatr Nurs. (2018) 9:170–4. doi: 10.14744/phd.2018.93723

[24] Yuksel R Yaylaci E Kaya H Erzin G Akdag E Demirci A. Sexual functions and prolactin levels in patients with bipolar disorder. Turkish J Clin Psychiatry. (2019) 22:48–56. doi: 10.5505/kpd.2019.03521

[25] Hariri AG Karadag F Gurol DT Aksoy UM Tezcan AE. Sexual problems in a sample of the Turkish psychiatric population. Compr Psychiatry. (2009) 50:353–60. doi: 10.1016/j.comppsych.2008.09.009 19486734

[26] Ghormode D Gupta P Ratnani D Aneja J. Evaluation of sexual dysfunction and quality of life in patients with severe mental illness: A cross-sectional study from a tertiary care center in Chhattisgarh. Ind Psychiatry J. (2019) 28:75–81. doi: 10.4103/ipj.ipj_16_19 31879451 PMC6929216

[27] Aldemir E. Akdeniz F. Isikli S. Keskinoz Bilen N. Vahip S.. (2016) Reproductive and Sexual Functions in Bipolar Patients: Data from a Specialized Mood Disorder Clinic. Dusunen Adam: J Psychiatry Neurological Sci. 29(1). doi: 10.5350/DAJPN2016290107

[28] Nagaraj AK Nizamie HS Akhtar S Sinha BN Goyal N. A comparative study of sexual dysfunction due to typical and atypical antipsychotics in remitted bipolar-I disorder. Indian J Psychiatry. (2004) 46:261–6. PMC2951652 21224908

[29] Phillips RL Jr Slaughter JR. Depression and sexual desire. Am Fam Physician. (2000) 62:782–6. 10969857

[30] Serretti A Chiesa A. Treatment-emergent sexual dysfunction related to antidepressants: a meta-analysis. J Clin Psychopharmacol. (2009) 29:259–66. doi: 10.1097/JCP.0b013e3181a5233f 19440080

[31] Grover S Ghosh A Sarkar S Chakrabarti S Avasthi A. Sexual dysfunction in clinically stable patients with bipolar disorder receiving lithium. J Clin Psychopharmacol. (2014) 34:475–82. doi: 10.1097/JCP.0000000000000131 24781439

[32] Lam D Donaldson C Brown Y Malliaris Y. Burden and marital and sexual satisfaction in the partners of bipolar patients. Bipolar Disord. (2005) 7:431–40. doi: 10.1111/j.1399-5618.2005.00240.x 16176436

[33] Wildes JE Marcus MD Fagiolini A. Prevalence and correlates of eating disorder co-morbidity in patients with bipolar disorder. Psychiatry Res. (2008) 161:51–8. doi: 10.1016/j.psychres.2007.09.003 PMC2643248 18782643

[34] McElroy SL Frye MA Suppes T Dhavale D Keck PE Jr Leverich GS. Correlates of overweight and obesity in 644 patients with bipolar disorder. J Clin Psychiatry. (2002) 63:207–13. doi: 10.4088/jcp.v63n0306 11926719

[35] Seixas C Miranda-Scippa A Nery-Fernandes F Andrade-Nascimento M Quarantini LC Kapczinski F. Prevalence and clinical impact of eating disorders in bipolar patients. Braz J Psychiatry. (2012) 34:66–70. doi: 10.1016/S1516-4446(12)70012-0 22392391

[36] Ruiz EMÁ. Gutiérrez-Rojas L. Comorbidity of bipolar disorder and eating disorders. Rev Psiquiatría y Salud Ment (English Edition). (2015) 8:232–41. doi: 10.1016/j.rpsmen.2015.05.001 25745820

[37] McDonald CE Rossell SL Phillipou A. The comorbidity of eating disorders in bipolar disorder and associated clinical correlates characterised by emotion dysregulation and impulsivity: A systematic review. J Affect Disord. (2019) 259:228–43. doi: 10.1016/j.jad.2019.08.070 31446385

[38] Ramacciotti CE Paoli RA Marcacci G Piccinni A Burgalassi A Dell’Osso L. Relationship between bipolar illness and binge-eating disorders. Psychiatry Res. (2005) 135:165–70. doi: 10.1016/j.psychres.2004.04.014 15922456

[39] Al-Halabi S Garcia-Portilla MP Saiz PA Fonseca E Bobes-Bascaran MT Galván G. Psychometric properties of the Spanish version of the Body Weight, Image and Self-Esteem Evaluation Questionnaire in patients with severe mental disorders. Compr Psychiatry. (2012) 53:1237–42. doi: 10.1016/j.comppsych.2012.04.001 22578984

[40] Castrogiovanni S Soreca I Troiani D Mauri M. Binge eating, weight gain and psychosocial adjustment in patients with bipolar disorder. Psychiatry Res. (2009) 169:88–90. doi: 10.1016/j.psychres.2008.06.016 19625088

[41] Kadioglu P Yetkin DO Sanli O Yalin AS Onem K Kadioglu A. Obesity might not be a risk factor for female sexual dysfunction. BJU Int. (2010) 106:1357–61. doi: 10.1111/j.1464-410X.2010.09348.x 20394615

[42] Kiliçlar M. The relationship between sexual satisfaction and body image and self-esteem. İstanbul: Işık University (2018).

[43] Davison TE McCabe MP. Relationships between men’s and women’s body image and their psychological, social, and sexual functioning. Sex Roles. (2005) 52:463–75. doi: 10.1007/s11199-005-3712-z

[44] Quinn-Nilas C Benson L Milhausen RR Buchholz AC Goncalves M. The relationship between body image and domains of sexual functioning among heterosexual, emerging adult women. Sex Med. (2016) 4:e182–9. doi: 10.1016/j.esxm.2016.02.004 PMC5005305 27036088

[45] Seal BN Meston CM. The impact of body awareness on sexual arousal in women with sexual dysfunction. J Sex Med. (2007) 4:990–1000. doi: 10.1111/j.1743-6109.2007.00525.x 17627744 PMC2859459

[46] Kim JS Kang S. A study on body image, sexual quality of life, depression, and quality of life in middle-aged adults. Asian Nurs Res (Korean Soc Nurs Sci). (2015) 9:96–103. doi: 10.1016/j.anr.2014.12.001 26160236

[47] Scheffers M van Busschbach JT Bosscher RJ Aerts LC Wiersma D Schoevers RA. Body image in patients with mental disorders: Characteristics, associations with diagnosis and treatment outcome. Compr Psychiatry. (2017) 74:53–60. doi: 10.1016/j.comppsych.2017.01.004 28095340

[48] Duggan JM Toste JR Heath NL. An examination of the relationship between body image factors and non-suicidal self-injury in young adults: the mediating influence of emotion dysregulation. Psychiatry Res. (2013) 206:256–64. doi: 10.1016/j.psychres.2012.11.016 23219105

